# The impact of glucagon-like peptide-1 (GLP-1) agonists in the treatment of eating disorders: a systematic review and meta-analysis

**DOI:** 10.1007/s40519-025-01720-9

**Published:** 2025-02-01

**Authors:** Hanieh Radkhah, Shiva Rahimipour Anaraki, Peyvand Parhizkar Roudsari, Razman Arabzadeh Bahri, Diar Zooravar, Sara Asgarian, Reza Hosseini Dolama, Ali Alirezaei, Razieh Khalooeifard

**Affiliations:** 1 Sina Hospital, Department of Internal Medicine, Tehran University of Medical Sciences, Tehran, Iran; 2 School of Medicine, Iran University of Medical Sciences, Tehran, Iran; 3 Tehran University of Medical Sciences, Tehran, Iran; 4 School of Medicine, Tehran University of Medical Sciences, Tehran, Iran; 5 Cellular and Molecular Research Center, Research Institute for Endocrine Sciences, Shahid Beheshti University of Medical Sciences, Tehran, Iran; 6 Department of Clinical Nutrition School of Nutritional Sciences and Dietetics, Tehran University of Medical Sciences, Tehran, Iran; 7 Students’ Scientific Research Center (SSRC), Tehran University of Medical Sciences, Tehran, Iran; 8 Tehran Heart Center, Cardiovascular Diseases Research Institute, Tehran University of Medical Sciences, Tehran, Iran; 9 Assistant Professor of Clinical Nutrition, Department of Clinical Nutrition, School of Nutritional Sciences and Dietetics, Tehran University of Medical Sciences, Tehran, Iran

**Keywords:** Glucagon-like peptide 1, GLP-1, Eating disorders, Meta-analysis

## Abstract

**Purpose:**

Glucagon-like peptide-1 (GLP-1) receptor agonists have shown potential in managing eating disorders (EDs). Recent studies highlight their effects on pathophysiological pathways, indicating their therapeutic promise, particularly for binge eating disorder (BED). This systematic review evaluates the therapeutic effects of GLP-1 agonists on BED, focusing on weight management and eating behaviors.

**Methods:**

A systematic search of PubMed, Scopus, Web of Science, and the Cochrane Library, along with manual searches, identified studies assessing GLP-1 agonists in BED patients up to November 8, 2024. Observational studies and clinical trials meeting inclusion criteria were analyzed.

**Results:**

Five studies (182 participants) were included. Patients receiving GLP-1 agonists experienced greater weight loss (− 3.81 kg; 95% CI − 5.14 to − 2.49; *p* < 0.01, *I*^2^: 59.88%) compared to controls. GLP-1 agonists significantly reduced BMI (− 1.48 kg/m^2^) and waist circumference (− 3.14 cm). Binge Eating Scale (BES) scores improved significantly (− 8.14 points; 95% CI − 13.13 to − 3.15; *p* < 0.01), though heterogeneity was noted.

**Conclusions:**

This review underscores the potential role of GLP-1 agonists in BED management. However, given the limited data, especially concerning EDs other than BED and the long-term effects of these medications, further comprehensive clinical trials are recommended to evaluate the impact of various GLP-1 agonists on different EDs across diverse demographic groups.

**Level of evidence:**

Level I, randomized controlled trials.

**Supplementary Information:**

The online version contains supplementary material available at 10.1007/s40519-025-01720-9.

## Points

1. Weight management benefits: GLP-1 agonists significantly enhance weight loss and reduce BMI and waist circumference in BED patients.
2. Mechanistic insights: These medications influence central and peripheral pathways, improving satiety and reducing binge-eating episodes through brain–gut interactions and modulation of reward systems.
3. Potential side effects: Nausea, vomiting, and other gastrointestinal symptoms may complicate ED treatment; clinicians must weigh these risks against benefits and discuss them with patients.
4. Research gaps: Evidence is limited for EDs other than BED. Long-term studies are needed to confirm safety, efficacy, and broader therapeutic applications.

**Supplementary Information:**

The online version contains supplementary material available at 10.1007/s40519-025-01720-9.

## Introduction

Eating disorders (EDs) are serious public health issues associated with reduced quality of life, increased healthcare utilization, and high rates of psychiatric comorbidities. Among EDs, binge eating disorder (BED) and bulimia nervosa (BN) are also linked to obesity and related conditions, including type 2 diabetes mellitus (T2DM) and cardiovascular disease [1]. BED is also the most prevalent ED, highlighting it as a significant concern [2]. BED is characterized by episodes of consuming large quantities of food, often accompanied by a sense of loss of control, guilt, and distress. It is frequently associated with psychological factors, such as depression and anxiety [3]. Despite the substantial public health impact of EDs, few effective medical treatments are currently available [4]. Lisdexamfetamine (LDX) and topiramate have been administered on a limited basis as treatments for BED [5, 6]. However, the effectiveness of these treatments has been restricted by significant side effects [5, 7]. Recently, glucagon-like peptide-1 (GLP-1) analogs have shown promise in targeting pathophysiological pathways implicated in BED.

GLP-1 analogs have demonstrated various beneficial pharmacological effects, with their most significant actions observed in managing T2DM. These effects include β-cell proliferation, increased insulin secretion, weight loss, and glucose-dependent insulinotropic and glucagonostatic functions [8, 9]. However, the effects of GLP-1 analogs extend beyond T2DM, offering a range of physiological benefits, including neuroprotection, improved cognitive function, cardiac benefits, reduced blood pressure, suppressed acid secretion, enhanced lipolysis, and modulation of inflammation [10].

GLP-1 plays a key role in central satiety signaling and is known to delay gastric emptying, enhance satiety, and promote weight reduction in patients with DM [11]. GLP-1, an anorexigenic hormone, is secreted by enteroendocrine cells in the gastrointestinal tract and by preproglucagon neurons in the brain, which together constitute the peripheral and central components of the GLP-1 system. These systems act independently to suppress appetite via distinct gut–brain circuits [12]. In rodent studies, GLP-1 receptors are present in key areas of the central nervous system that regulate appetite. GLP-1 analogs have been shown to modulate neurotransmitter release related to hunger and reward in the hypothalamus and striatum, resulting in reduced food intake [13]. GLP-1 receptor signaling is essential for regulating both homeostatic feeding behavior and non-homeostatic control of food reward. The neural pathways involved in GLP-1 receptor-mediated reduction in food intake include feeding centers in the hypothalamus and hindbrain, the mesolimbic reward system (such as the ventral hippocampus), and certain forebrain regions, including the medial prefrontal cortex [14, 15].

Several studies have highlighted the significant benefits of GLP-1 analogs in managing EDs and obesity across multiple domains [16, 17]. For example, in individuals with obesity undergoing behavioral weight loss interventions, liraglutide administration led to more substantial reductions in global ED psychopathology, shape concerns, and dietary disinhibition compared to those receiving only behavioral weight loss interventions [17, 18]. These findings and the underlying pathophysiological mechanisms suggest that GLP-1 analogs hold promise as a potential therapeutic approach for managing obesity and EDs [19].

Given the importance of identifying new therapies for BED and the promising outcomes associated with GLP-1 receptor agonists, this article systematically reviews the effects of these drugs on BED, with a specific focus on their impact on anthropometric variables. Increasing clinician awareness of the therapeutic potential of GLP-1 analogs may improve management strategies for patients with BED.

## Methods

### Protocol and registration

This systematic review and meta-analysis was conducted according to the preferred reporting items for systematic reviews and meta-analyses (PRISMA) guidelines [20]. The PICO model was applied in this study. The acronym PICO stands for population (individuals with BED), intervention (administration of GLP-1 agonists in individuals with BED), comparison (individuals with BED who received a placebo, lifestyle interventions, and other medications excluding GLP-1 agonists), and outcomes (effectiveness of GLP-1 agonists, particularly about weight loss) as reported in the articles.

The primary outcome of this study was to evaluate the impact of GLP-1 agonists on weight loss, body mass index and waist circumference in patients with BED. The secondary outcome included the impact of GLP-1 agonists on emotional eating behaviors including emotional eating scores. The study protocol was reviewed and confirmed by the Ethics Committee of Tehran University of Medical Sciences (TUMS), with the following registration label: IR.TUMS.SINAHOSPITAL.REC.1.1403.176. The primary outcome was the impact of GLP-1 agonists on weight loss, including BMI, weight, and WC. The secondary outcomes included the effects on emotional eating behaviors, such as emotional eating scores.

### Search strategy and databases

Two researchers independently searched four databases—PubMed, Scopus, Web of Science, and the Cochrane Library—and retrieved all eligible articles published up to November 8, 2024. The search strategy for each database is presented in Table S1. In addition, a manual search was conducted using the references of the selected articles and Google Scholar to ensure the inclusion of relevant studies.

### Eligibility criteria

Studies with the following criteria were included: (1) population/problem: original articles, including observational studies and clinical trials assessing people with EDs with no restriction on age and gender; (2) intervention: studies assessing the impact of GLP-1 agonists with no restriction on route and dosage or using other types of antidiabetic medications; (3) comparison (control): assessing a group of patients as control group not receiving GLP-1 agonists (including placebo, other medications, lifestyle or diet intervention); and (4) outcome: assessing the impact of GLP-1 agonists on weight loss [including body mass index (BMI), weight and waist circumference (WC)] as primary outcome and emotional eating behaviors (including emotional eating scores) as secondary outcome.

The exclusion criteria were as follows: (1) not assessing EDs; (2) not administrating GLP-1 agonist; (3) reviews, case reports and study protocols; and (4) inaccessible full text or insufficient data.

### Screening and data extraction

After removing duplicate studies from the initial search results, two authors independently screened the titles and abstracts to exclude irrelevant articles. The full text of the selected articles were then reviewed by two independent researchers, examining study design, interventions, and outcomes in detail to determine eligibility. Any inconsistencies were resolved through group discussions in online meetings, facilitated by H.R. until a consensus was reached. Data extraction began by generating a structured database containing each article's title, authors, and publication date. Subsequently, two authors independently extracted the following detailed information from the included studies: sample size, study design, type of GLP-1 agonist, follow-up period, and participants’ weight, BMI, and eating disorder scores before and after the intervention.

### Quality assessment and risk of bias

The quality of the studies was assessed using appropriate tools based on the study design. For clinical trials, the Cochrane Risk of Bias (RoB) tool was employed, whereas the STROBE checklist was used for observational studies, such as Richard et al.'s study [21, 22]. Two investigators independently assessed study quality, compared results, and resolved any inconsistencies through discussion until reaching a consensus.

### Data synthesis and statistical analysis

Quantitative variables were reported as means with standard deviations (SDs) or median (interquartile range (IQR)). We used the methods of Wan et al. and Lou et al. to estimate the mean (SD) using the median (IQR) and sample size [23, 24]. Weighted mean difference (WMD) was conducted to compare the mean (SD) changes of anthropometric variables and BES score in the intervention and comparison group, as they were all reported on the same scale. We used the random effects model to consider heterogeneity between studies. In addition, we calculated I^2^ statistics and *p* value from Cochran’s Q test to measure heterogeneity among studies. A *p* value less than 0.05 was considered a significant heterogeneity. Subgroup analysis and Galbraith (radial) plot were used to find the source of heterogeneity in studies with significant heterogeneity. Publication bias was assessed using Egger’s test. All statistical analyses were performed using STATA statistical software, version 17 (StataCorp, College Station, TX, USA). A *p* value *of* < *0.05* was considered a significant finding.

## Results

### Identification of studies

**Fig. 1:**
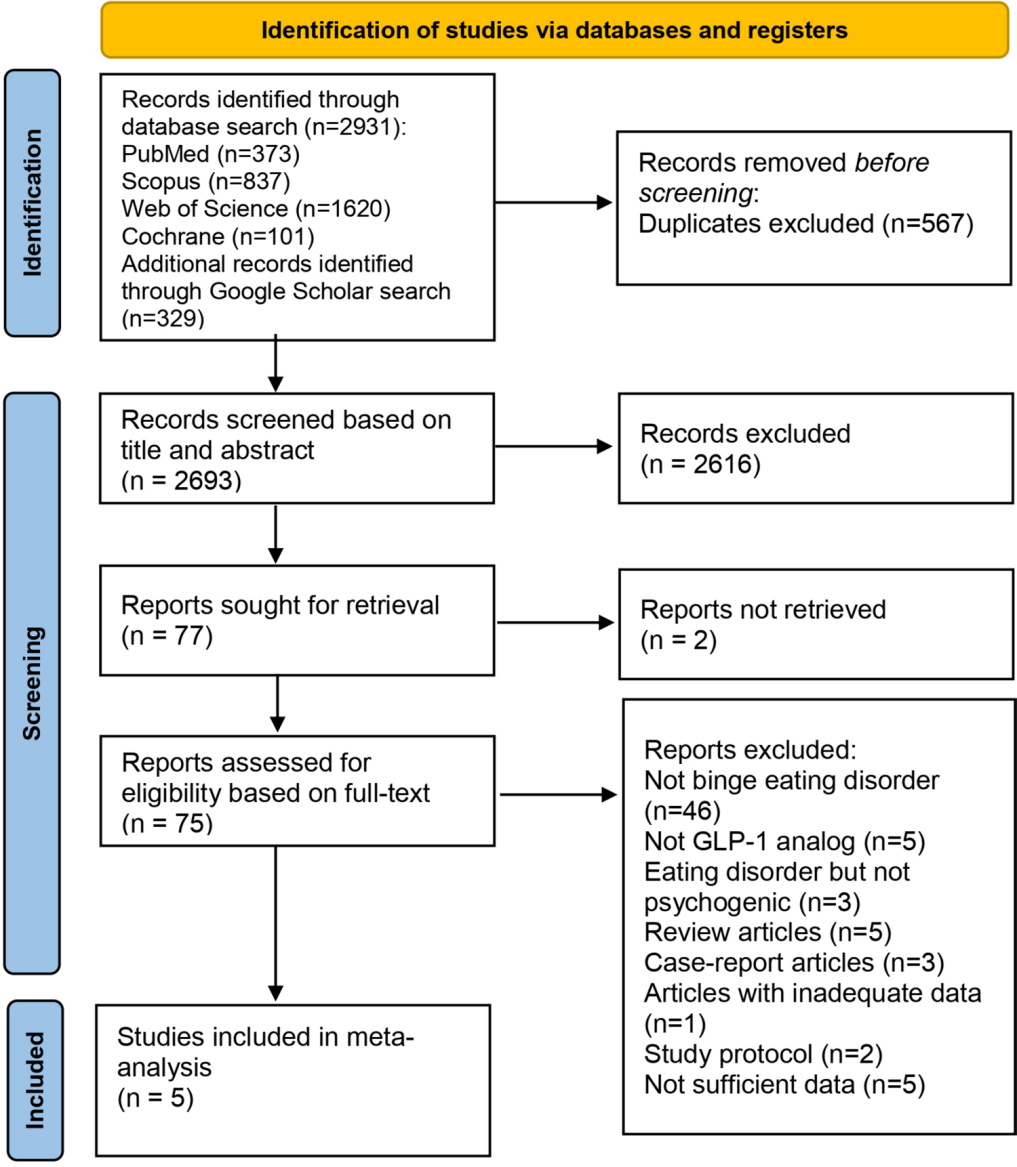
PRISMA flowchart

2931 articles were retrieved from international database searches, including the Cochrane Library, Web of Science, PubMed, and Scopus. An additional 329 articles were identified through Google Scholar searches. After removing duplicates, 2693 articles remained for screening based on titles and abstracts. A total of 2616 studies were excluded according to the eligibility criteria. As a result, 75 full-text articles were reviewed, and 5 studies were included in the final meta-analysis [11, 18, 25–27] (Fig. 1).

### Study characteristics

The characteristics of the studies included in the meta-analysis are presented in Table 1. These studies were published in 2015 and 2023. A total of 182 patients were assessed across the studies, with 91 patients in the case group receiving GLP-1 agonists and 91 patients in the control group. Three studies were conducted in the United States, and two studies were conducted in Ukraine and Italy.

**Table 1:** Description of the studies

First author, year	Type of study	Intervention/control	Female %	Age	Number for intervention/control	Duration of intervention	Weight change (Kg)	BMI change (Kg/m2)	WC change (cm)	BES score change
Allison et al. 2023	Randomized double-blind controlled trial	Liraglutide (3 mg)	46.1%	46.3 ± 7.8	(*n* = 13/7 completed)	17 weeks	− 4.7 ± 2.88	− 1.3 ± 1.44	− 4 ± 3.97	NR
		Placebo	78.5%	42.8 ± 12.5	(*n* = 14/7 completed)		− 0.9 ± 2.62	− 0.3 ± 1.49	− 1.2 ± 3.67	NR
Richard et al. 2023	Open-label retrospective cohort study	Semaglutide	89.4%	43.5 [13.6, 22–74]	*n* = 19	Average of change in 180 days	− 10.2 ± 6.48	NR	NR	− 14 ± 8.2
		alternative anti-obesity medications but not receiving semaglutide	87.5%	39.6 [12.8, 21–67]	*n* = 16		− 13.2 [20.1, − 23.54–67.1]	NR	NR	− 5.9 ± 9.1
Lanius et al. 2022	Abstract, trial	Metformin and liraglutide 1.8 mg daily -12 weeks	NR		*n* = 8	12 weeks	− 4.3 ± 1.3	NR	NR	NR
		Metformin and SGLT-2 inhibitors			*n* = 10		− 1.7 ± 0.8	NR	NR	NR
Robert et al. 2015	Randomized, prospective, controlled trial	Liraglutide 1.8 mg daily, exercise and diet	NR		*n* = 21	12 weeks	− 4.4 ± 8.59	− 1.75 ± 2.12	− 3.71 ± 6.2	− 10.44 ± 3.19
		Exercise and diet			*n* = 21		− 0.76 ± 7.11	− 0.76 ± 2.36	− 0.25 ± 2.36	− 6.15 ± 3.55
Da Porto et al. 2020	Pilot open-label, prospective controlled study	Dulaglutide	47.7%	54,2 ± 8,9	*n* = 30	12 weeks	− 4.53 ± 2.17	− 1.65 ± 0.81	NR	− 11.93 ± 7.11
		Gliclazide	60%	55,1 ± 6,433	*n* = 30		0.49 ± 1.91	0.1 ± 0.77	NR	0.3 ± 3.23

BED: Binge eating disorder; BES: Binge eating scale

### Meta-analysis

**Fig. 2:**
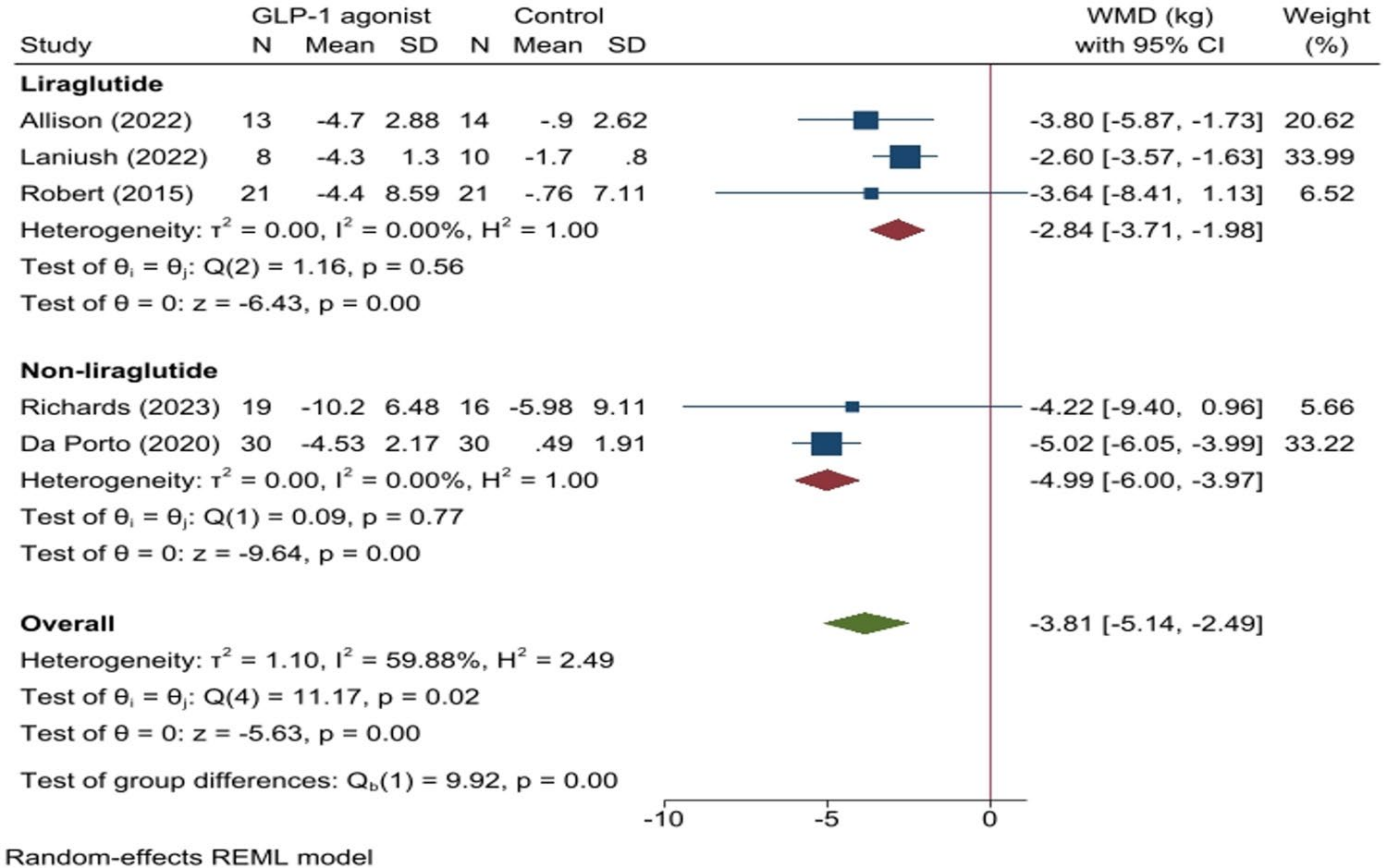
Forest plot for analysis comparing the weight changes (kg) in patients with BED administrated with GLP-1 agonist (GLP-1 agonist) to controls (control) at follow-up. This forest plot presents the weighted mean difference (WMD) with 95% confidence intervals (CI) for weight changes (kg) in patients with BED administrated with GLP-1 agonist (GLP-1 agonist) compared to controls (control). Results are categorized by the type of GLP-1 agonist: liraglutide and non-liraglutide (Semaglutide and Dulaglutide). Each study’s sample size (N), mean, and standard deviation (SD) for treatment and control groups are shown. The diamond shapes represent pooled estimates, with the overall effect size at the bottom. Heterogeneity statistics and p-values for the test of group differences are also provided

**Fig. 3:**
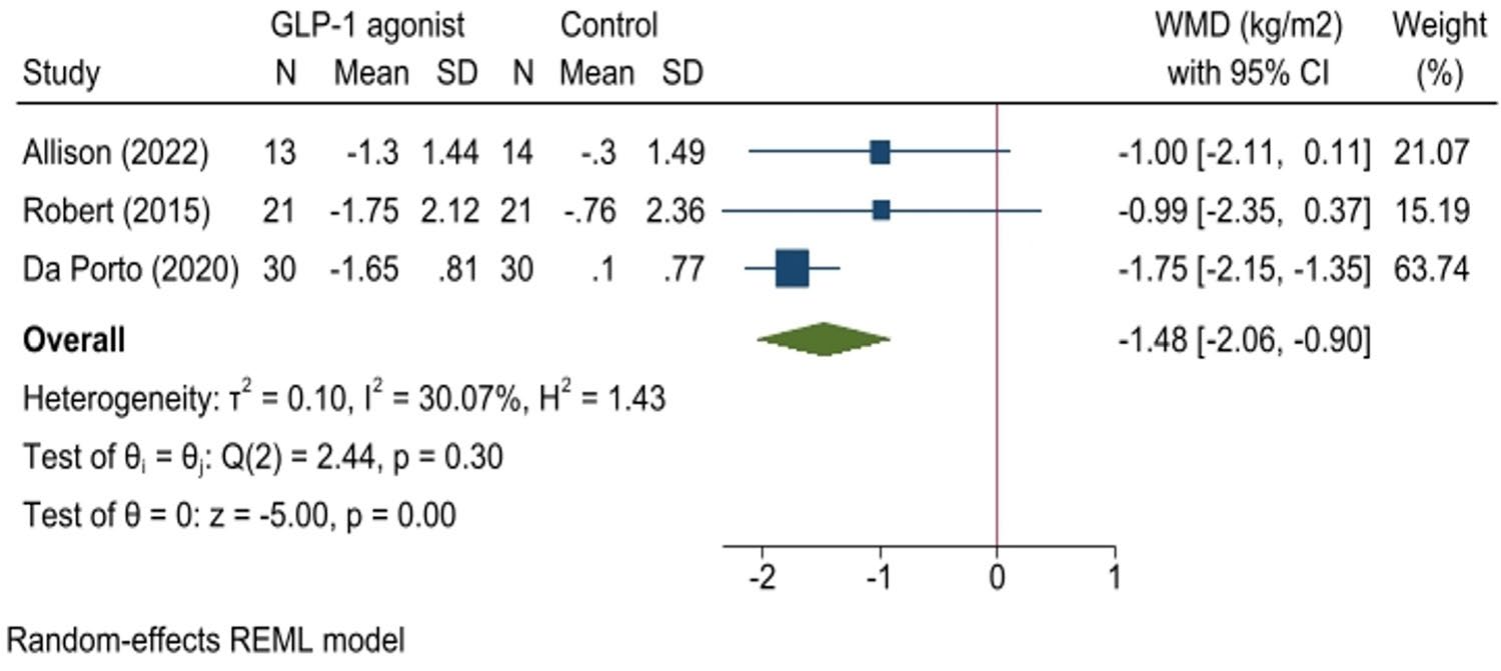
Forest plot for analysis comparing the BMI changes (kg/m^2^) in patients with BED administrated with GLP-1 agonist (GLP-1 agonist) to controls (control) at follow-up. This forest plot presents the weighted mean difference (WMD) with 95% confidence intervals (CI) for BMI changes (kg/m^2^) in patients with BED administrated with GLP-1 agonist (GLP-1 agonist) compared to controls (control). Each study’s sample size (N), mean, and standard deviation (SD) for treatment and control groups are shown. The diamond shapes represent pooled estimates, with the overall effect size at the bottom. Heterogeneity statistics and p-values are also provided

**Fig. 4:**
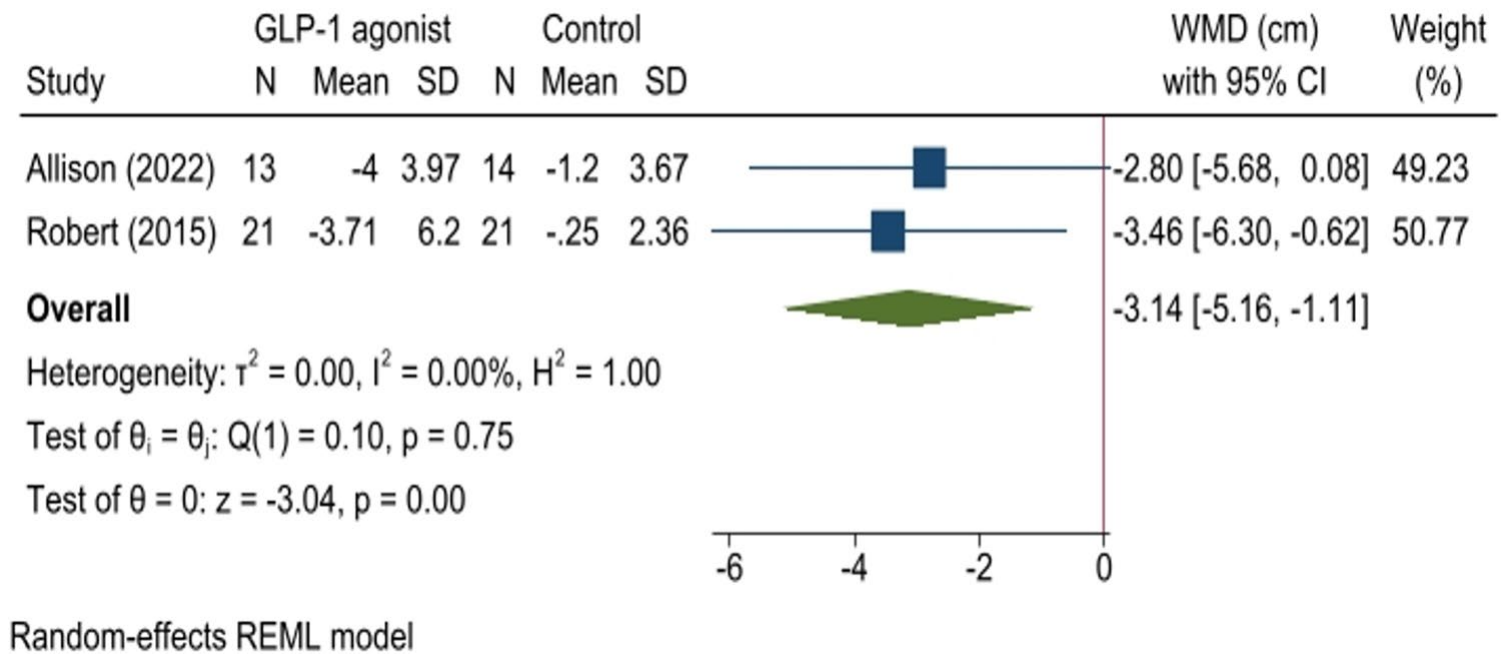
Forest plot for analysis comparing the WC changes (cm) in patients with BED administrated with GLP-1 agonist (GLP-1 agonist) to controls (control) at follow-up. This forest plot presents the weighted mean difference (WMD) with 95% confidence intervals (CI) for WC changes (cm) in patients with BED administrated with GLP-1 agonist (GLP-1 agonist) compared to controls (control). Each study’s sample size (N), mean, and standard deviation (SD) for treatment and control groups are shown. The diamond shapes represent pooled estimates, with the overall effect size at the bottom. Heterogeneity statistics and p-values are also provided

The analysis included data from five studies examining the impact of GLP-1 agonists on anthropometric variables (weight, BMI, and WC) and eating behavior compared to controls in patients with BED. A pooled analysis of these studies revealed that patients who received GLP-1 agonists experienced a significantly greater weight loss of 3.81 kg (WMD: − 3.81, 95% CI − 5.14 to − 2.49, *p* < *0.01*, *I*^2^: 59.88%, Fig. 2) compared to comparators. Subgroup analysis indicated greater weight loss in patients treated with dulaglutide and semaglutide compared to those treated with liraglutide **(**Fig. 2**)**. Egger's test suggested no publication bias among the studies (*p* = *0.934*). In addition, the analysis showed that GLP-1 agonists significantly reduced BMI by 1.48 kg/m^2^ (WMD: − 1.48, 95% CI − 2.06 to − 0.90, *p* < *0.01*, *I*^2^: 30%, Fig. 3) and WC by 3.14 cm (WMD: − 3.14, 95% CI − 5.16 to − 1.11, *p* < *0.01*, *I*^2^: 0.0%, Fig. 4) compared to controls. The low *I*^2^ values and non-significant *p* value indicated minimal heterogeneity.

**Fig. 5:**
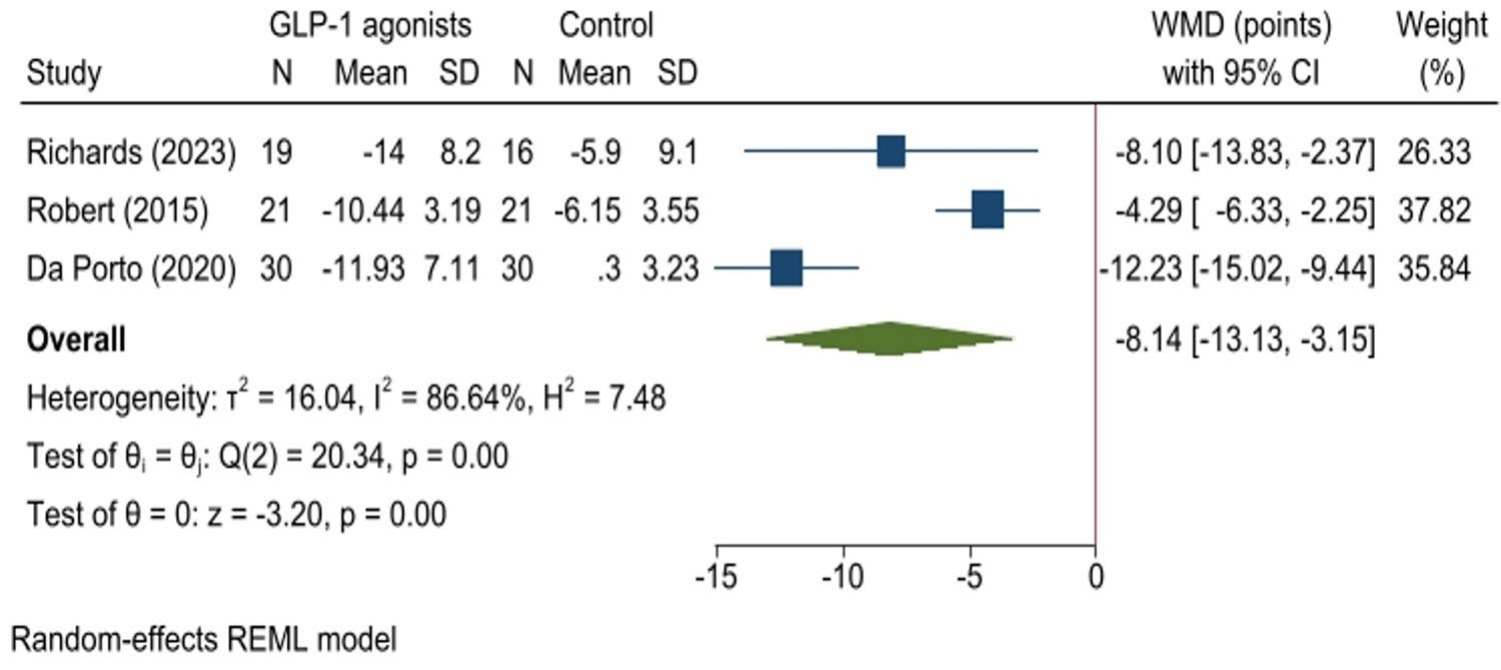
Forest plot for analysis comparing the BES score (points) in patients with BED administrated with GLP-1 agonist (GLP-1 agonist) to controls (control) at follow-up. This forest plot presents the weighted mean difference (WMD) with 95% confidence intervals (CI) for BES score (points) in patients with BED administrated with GLP-1 agonist (GLP-1 agonist) compared to controls (control). Each study’s sample size (N), mean, and standard deviation (SD) for treatment and control groups are shown. The diamond shapes represent pooled estimates, with the overall effect size at the bottom. Heterogeneity statistics and *p*-values are also provided

Furthermore, individuals treated with GLP-1 agonists showed a reduction in BES scores that was 8.14 points greater compared to the control group (WMD: − 8.14, 95% CI − 13.13 to − 3.15*, p* < *0.01*, *I*^2^: 86.64%, Fig. 5), indicating a statistically significant effect of GLP-1 agonists. Egger's test revealed no publication bias (*p* = *0.844*). Findings from the Galbraith plot did not identify any influential studies or outliers, further demonstrating the robustness of the results (Supplementary Fig. 1).

### Quality assessment of included studies

A study by Richard et al. showed moderate adherence to STROBE guidelines, with average compliance scores, indicating a mix of fair to good quality. Weaknesses of this study included incomplete reporting of bias mitigation strategies, unclear data sources, and limited discussion of generalizability. Clinical trials varied in risk of bias profiles. While, some achieved low risk across all domains and were classified as good quality, others exhibited unclear or high risks in areas, such as allocation concealment and blinding of participants or outcome assessors, resulting in fair or poor quality ratings. Despite these limitations, the majority of studies provided reliable data, with no critical risks that would invalidate their findings.

## Discussion

This is a systematic review and meta-analysis of the beneficial effects of GLP-1 analogs on eating disorders, with a particular emphasis on anthropometric variables and eating behaviors in BED.

We noticed that those with BED who received GLP-1 analogs lost 3.81 kg more weight than comparators, which was a significantly greater amount. A recent systematic review and meta-analysis observed that adults with obesity or overweight who were administered GLP-1 analogs for at least 12 weeks, showed a significant overall improvement in excess weight loss when compared to a placebo, with a weighted mean difference of 3.11 kg [28].

The DSM-5 includes the latest criteria for defining EDs, emphasizing behavioral indicators rather than physical or cognitive criteria. The primary EDs described are anorexia nervosa (AN), bulimia nervosa (BN), binge-eating disorder (BED), avoidant/restrictive food intake disorder (ARFID), and other specified feeding or eating disorders [29]. Despite the serious health consequences of EDs including malnutrition, cardiac disease, and gastrointestinal complications, their global prevalence continues to rise, with more individuals diagnosed each year [30]. A significant correlation exists between abnormal eating patterns and the development of EDs. For instance, binge-eating episodes, marked by excessive food intake and a loss of control over eating behavior, are characteristic of BED, BN, and the binge-purge subtype of anorexia nervosa (AN-BP) [31]. Emotional eating (EE), a tendency to overeat in response to negative emotions, also shows notable similarities to BED, as both involve difficulties with inhibition and emotion regulation [32]. Early intervention in individuals exhibiting emotional eating behaviors may prevent these patterns from progressing into full EDs [33]. Consequently, further research is essential to better understand the range of problematic eating patterns not currently included in diagnostic manuals [29].

Eating behavior is not passively determined by hormonal fluctuations and energy demands [34]. Instead, it is a complex behavior influenced by multiple factors, including central reward processing, food hedonic value, gut-originated reward pathways, gut microbiota environment, genetics, cognition, and psychological factors, such as anxiety and depression [35]. Specific brain regions regulate energy intake by the body's metabolic state. The hypothalamus serves as the center of the homeostatic system and includes the arcuate nucleus, ventromedial and lateral hypothalamus, and reward systems (amygdala, hippocampus, and nucleus accumbens [NAc]). Behavior is modified to obtain specific foods, and cognitive modulation enhances the adaptability of these behaviors. Consequently, aberrant eating behaviors, such as binge eating and food avoidance, often result from deficits in inhibitory control and other cognitive functions [34]. Notably, ED exhibits neurobiological mechanisms comparable to those in substance use disorders (SUD). In response to palatable foods and illicit substances, similar brain regions and neurotransmitter systems become activated [36]. Distorted cognitive function, a hallmark of substance use disorders, is also associated with maladaptive eating behaviors [34, 37]. Furthermore, genome-wide association (GWAS) and epigenetic studies have identified multiple genetic polymorphisms associated with ED, including variations in the glucocorticoid receptor gene, dopamine receptor gene, μ-opioid receptor gene, 5HT transporter gene, and GF-II isoforms [38, 39]. Many of these genes are also implicated in substance use disorders and BED, particularly those regulating serotonin (5-HT) and dopamine (DA) in the central nervous system [40, 41]. In summary, eating behaviors are influenced by a range of biological, psychological, and genetic factors, with disruptions contributing to disorders like binge eating.

Optimistically, the complex and multifactorial mechanisms underlying eating behavior present numerous potential therapeutic targets. Psychological interventions currently represent the primary treatment option for patients with EDs [30, 42]. Only two drugs are approved by the FDA for the treatment of EDs: LDX for moderate and severe binge eating in adults aged 18 to 55 and fluoxetine for BN [43]. However, several pharmaceutical medications have shown promise in clinical trials for treating specific EDs. For example, a variety of options have showed efficiency for treating BED, including antidepressants, anticonvulsants, opioid receptor antagonists, and anti-obesity medicines, such as cannabinoid receptor 1 (CB1) antagonists and GLP-1 analogs [44].

GLP-1 analogs were initially approved for the treatment of T2DM [44–46]. Subsequently, research identified the role of GLP-1 in appetite regulation and body weight control, mediated by a complex interaction between the brain and the gut [45]. Numerous systematic reviews have documented the efficacy of various GLP-1 analog classes in promoting weight loss [38, 47, 48]. Based on these findings, the FDA approved Semaglutide and Liraglutide as anti-obesity drugs [49]. The appetite-suppressing and gastrointestinal motility-reducing effects of GLP-1 analogs, along with side effects, such as nausea, vomiting, and diarrhea, may contribute to dietary disinhibition. This connection is considered one of the mechanisms through which GLP-1 analogs support the treatment of specific EDs including BED [50]. This study identified a correlation between the administration of GLP-1 analogs and increased dietary disinhibition in this context. In addition, GLP-1 and its receptors have been located in specific brain regions, particularly in the brainstem and hypothalamus [51]. Neurons in the nucleus of the solitary tract produce GLP-1, and both GLP-1 and its analogs can cross the blood–brain barrier (BBB). Thus, researchers have examined the central effects of GLP-1 analogs [52], and multiple studies have shown these medications to have beneficial cognitive effects [52, 53]. This study also demonstrated a positive correlation between treatment with GLP-1 analog and enhanced cognitive restraint scores, suggesting potential therapeutic implications for GLP-1 analogs in specified EDs management. Furthermore, the expression of GLP-1 components within the mesolimbic reward circuitry suggests that GLP-1 influences brain reward systems. This finding has led to studies showing that GLP-1 analogs may positively impact conditions associated with impaired reward systems, such as problematic food intake and addictions to alcohol, nicotine, and other drugs [54–56]. Consequently, modulation of the reward system represents an additional mechanism by which GLP-1 analogs may be beneficial in treating specific EDs, such as BED.

Regarding the effects of GLP-1 analogs on EDs, most available data focuses on BED. However, based on studies examining other eating patterns and the shared mechanisms underlying various EDs, the beneficial effects of GLP-1 analogs likely extend beyond BED. One case report described a patient with polycystic ovarian syndrome (PCOS) receiving long-term metformin treatment; the patient had depression, bulimia nervosa, and reactive hypoglycemia, and was unresponsive to psychotherapy or antidepressants. The patient claimed that her bulimic behavior completely disappeared in 2 weeks after adding liraglutide to the metformin, and she remained asymptomatic for 5 years while receiving metformin and liraglutide [57]. Another case report described a patient with autism and compulsive eating behaviors who responded well to liraglutide [58]. In addition, multiple studies have demonstrated the efficacy of GLP-1 analogs in modifying eating behaviors. In an observational study of 69 participants, semaglutide was highly effective in ameliorating EE and other abnormal eating patterns. After 3 months of semaglutide, the percentage of patients with EE decreased from 72.5 to 11.5%, the percentage of patients with external eating decreased from 27.5 to 10.1%, the percentage of patients with craving decreased from 49.3 to 21.7%, and the percentage of patients with savory craving decreased from 53.6 to 14.5% [59]. Another study, which included 34 participants with obesity and T2DM, assessed dietary changes using the Japan Society for the Study of Obesity questionnaire. Significant improvements in eating behavior scores were observed after 6 months of semaglutide treatment [60]. A recent study found that GLP-1 analogs improve the mental health of patients receiving treatment for type 2 diabetes and obesity. Because of the improvements in blood glucose control and weight, participants reported an overall favorable experience with the medicine; some also reported changes in mood, anxiety, confidence, self-esteem, and interpersonal relationships. In addition, many mentioned that their eating habits had changed [61].

This study conducted the first systematic review and meta-analysis on the efficacy of GLP-1 analogs in the treatment of EDs. The findings offer promising indications for the potential future application of GLP-1 analogs in managing EDs. To aid clinicians in making informed treatment decisions for individuals with EDs, it is essential to increase awareness of the therapeutic properties of GLP-1 analogs. A potential association exists between the therapeutic effects of GLP-1 analogs and improvements in eating patterns via multiple pathways. First, GLP-1 analogs contribute to appetite regulation and reduced dietary disinhibition through a complex brain–gut interaction that affects hormone production, postprandial gastric emptying, and intestinal motility. Second, evidence suggests that these medications modulate the mesolimbic system, leading to reduced responses to rewarding foods, lower intake of palatable foods, and decreased food addiction. Third, these analogs may enhance cognitive restraint related to eating behaviors. Notably, the potential adverse effects of GLP-1 analogs warrant careful consideration and should be further investigated in long-term studies that account for various EDs, GLP-1 analog types, and diverse demographic populations. These include the potential for medication misuse and other adverse effects. The majority of data on adverse effects of GLP-1 analogs were provided in the reviewed studies. In a study by Allison et al., serious adverse events occurred in the liraglutide group, including hospitalization for vomiting and laparoscopic cholecystectomy, with a higher incidence of adverse events in the liraglutide group compared to placebo group (89.5% vs. 64.7%)[18]. GLP-1 analogs may lead to dietary restriction by decreasing hunger and gastrointestinal upset [62]. There have also been reports of psychiatric adverse effects linked to GLP-1 analogs, such as anxiety, depression, suicidal behavior, eating disorders, fear of eating, and self-induced vomiting. Due to the significance of the negative consequences, the paucity of information, and inconsistent results, these findings should be considered and investigated in high-quality research with an extended follow-up period [63–66]. In addition, S. Bartel et al. suggest a risk of ED recurrence following treatment discontinuation or attenuation of medication effects over time, emphasizing the need for studies with extended follow-up periods [50].

This study had several limitations. First, the analysis was restricted by the small number of included studies. Second, it is important to acknowledge that the risk of bias assessment indicated moderate to high risk in several studies. Thus, the findings should be interpreted with caution.Third, the majority of data was focused on BED, and other EDs were not included in the final analysis. Given the shared underlying mechanisms of various EDs, it is essential to investigate the potential therapeutic effects of GLP-1 analogs for other EDs, particularly those associated with binge eating or EE. Fourth, due to limited data, crucial parameters linked to obesity or EDs, including laboratory biomarkers, could not be analyzed. Fourth, the small sample size may compromise the accuracy of the subgroup analysis. There is insufficient data on the effectiveness of GLP-1 analogs in distinct populations considering variables, such as gender, age, ethnicity, BMI, and comorbidities. Finally, the short follow-up periods in the available studies may have limited insights into their long-term effects. Future long-term clinical trials are required to address these limitations.

## Conclusion

The therapeutic effects of GLP-1 analogs on various diseases are well documented. This research emphasizes the importance of administering these medications to individuals with EDs, highlighting their potential benefits for weight loss. However, clinicians must also consider potential adverse effects, evaluate the cost-effectiveness of these medications for each patient, and closely monitor for any improvement or worsening of eating disorders and related side effects. Due to limited data on this topic, particularly concerning EDs other than BED, and the uncertain long-term impact of GLP-1 analogs on patients with EDs, comprehensive placebo-controlled studies are needed to evaluate the safety and efficacy of different GLP-1 analogs on diverse EDs across various demographic groups.

## Supplementary Information

Below is the link to the electronic supplementary material.

**Supplementary materials 1.**

**Supplementary materials 2.**

**Supplementary materials 3. Figure S1: **Gallbraith plot assessing the potential outliers among studies comparing BES score in patients receiving GLP-1 agonists compared to control group.

**Supplementary materials 4.**

## Data Availability

The data sets generated during and/or analyzed during the current study are available from the corresponding author upon reasonable request. No datasets were generated or analysed during the current study.
