# Supplementary material for: The impact of glucagon-like peptide-1 (GLP-1) agonists in the treatment of eating disorders: a systematic review and meta-analysis
Source: Eat Weight Disord. 2025 Feb 1;30(1):10. doi: 10.1007/s40519-025-01720-9 (PMC11787217; doi:10.1007/s40519-025-01720-9)
Supplement: Supplementary file 1 — Supplementary materials 1. [file 40519_2025_1720_MOESM1_ESM.docx]

Table S1. Search Strategy

| PubMed  (n=369) | 1. (("Glucagon-Like Peptide 1"[all] OR "Glucagon Like Peptide 1"[all] OR "GLP-1"[all] OR "GLP 1"[all] OR "Glucagon-Like Peptide-1"[all] OR "Liraglutide"[all] OR "Victoza"[all] OR "Saxenda"[all] OR "NN 2211"[all] OR "NN2211"[all] OR "NN-2211"[all] OR (2211[all] AND NN[all]) OR "exenatide"[all] OR "glucagonlike peptide-1 receptor"[all] OR "glucagon-like peptide-1 receptor agonist"[all] OR "lixisenatide"[all] OR "semaglutide"[all] OR "albiglutide"[all] OR "dulaglutide"[all] OR "Glucagon-Like Peptides"[all] OR "Glucagon Like Peptides"[all] OR (Polypeptide[all] AND "Glucagon-Like"[all]) OR "Glucagon-Like Polypeptides"[all] OR "Glucagon Like Polypeptide*"[all] OR (Peptide[all] AND "Glucagon-Like"[all]) OR "Gut Glucagon"[all] OR "Enteroglucagons"[all] OR (Glucagon[all] AND Gut[all]) OR "Glucagon-Like Peptide 2"[all] OR "Glucagon Like Peptide 2"[all] OR "Proglucagon (126-158)"[all] OR "Glucagon-Like Peptide-2 (1-33)"[all] OR "WB4-24"[all] OR "polyethylene glycol loxenatide"[all] OR "PEX168"[all] OR "GW002 peptide"[all] OR "rGLP-1"[all] OR "CNTO 736"[all] OR "CNTO-736"[all] OR "S 23521"[all] OR "(a8-des R36) GLP-1-(7-37)-NH2"[all] OR "Adlyxin"[all] OR "AQVE-10010"[all] OR "ZP10A peptide"[all] OR "ZP 10"[all] OR "ZP-10"[all] OR "glucagon-like peptide 1 (7-36)amide"[all] OR "GLP-1-(7-36) amide"[all] OR "GLP-1a"[all] OR "proglucagon (78-107) amide"[all] OR "GLP-I (7-36)NH2"[all] OR "glucagon-like peptide-I (7-36)amide"[all] OR "glucagon-like peptide-1(7-36 amide)"[all] OR "GLP-1 (7-36)amide"[all] OR "GLP-I (7-36)amide"[all] OR "MKC 253"[all] OR "MKC253"[all] OR "MKC-253"[all] OR "GLP-1(32-36)amide"[all] OR "BMS 686117"[all] OR "BMS686117"[all] OR "BMS-686117"[all] OR "Val(8)-"[all] OR "Val8-GLP-1(7-36)"[all] OR "LY 2189265"[all] OR "LY-2189265"[all] OR "LY2189265"[all] OR "Trulicity"[all] OR "Glucagon-Like Peptide-1 Receptor"[all] OR "Glucagon Like Peptide 1 Receptor"[all]) OR "GLP-1R Receptor"[all] OR "GLP 1R Receptor"[all] OR (Receptor[all] AND "GLP-1R"[all]) OR "GLP1R Protein"[all] OR (Protein[all] AND GLP1R[all]) OR "GLP-1 Receptor"[all] OR "GLP 1 Receptor"[all] OR (Receptor[all] AND "GLP-1"[all]) OR (Receptor[all] AND GLP1R[all]) OR "Glucagon-Like Peptide Receptors"[all] OR "Glucagon Like Peptide Receptors"[all] OR ("Peptide Receptors"[all] AND "Glucagon-Like"[all]) OR (Receptors[all] AND "Glucagon-Like Peptide"[all]) OR "GLPR Protein"[all] OR (Protein[all] AND "GLPR"[all]) OR "Glucagon-Like Receptor"[all] OR "Glucagon Like Receptor"[all] OR (Receptor[all] AND "Glucagon-Like"[all]) OR (Proteins[all] AND "GLPR"[all]) OR "Glucagon-Like Receptors"[all] OR "Glucagon Like Receptors"[all] OR (Receptors[all] AND "Glucagon-Like"[all]) OR "Glucagon-Like Peptide Receptor"[all] OR "Glucagon Like Peptide Receptor"[all] OR ("Peptide Receptor"[all] AND "Glucagon-Like"[all]) OR (Receptor[all] AND "Glucagon-Like Peptide"[all]) OR "Glucagon-Like Peptide-2 Receptor"[all] OR ("Peptide-2 Receptor"[all] AND "Glucagon-Like"[all]) OR (Receptor[all] AND "Glucagon-Like Peptide-2"[all]) OR "GLP-2 Receptor"[all] OR "GLP 2 Receptor"[all] OR (Receptor[all] AND "GLP-2"[all]) OR (Protein[all] AND "GLP2R"[all]) OR "Glucagon-Like Peptide 2 Receptor"[all] OR "Glucagon Like Peptide 2 Receptor"[all] OR (Receptor[all] AND "GLP-2R"[all]) OR "Pro-Glucagon"[all] OR "Pro Glucagon"[all] OR "Preproglucagons"[all] OR "Pre-Proglucagons"[all] OR "Glucagon Precursor*"[all])  AND  2. (("Feeding and Eating Disorders"[all] OR "Eating and Feeding Disorders"[all] OR (Disorder*[all] AND Feeding[all]) OR "Feeding Disorder*"[all] OR (Disorder*[all] AND Eating[all]) OR "Eating Disorder*"[all] OR "Appetite Disorders"[all] OR "Feeding and Eating Disorders of Childhood"[all] OR "Childhood Eating and Feeding Disorders"[all] OR "Food Addiction*"[all] OR "Compulsive Eating"[all] OR (Eating[all] AND Compulsive[all]) OR "Night Eating Syndrome*"[all] OR ("Eating Syndromes"[all] AND Night[all]) OR "Orthorexia Nervosa"[all] OR (Nervosa[all] AND Orthorexia[all]) OR "Orthorexia"[all] OR "Obsessive Healthy Eating"[all] OR ("Healthy Eating"[all] AND Obsessive[all]) OR "Pica"[all] OR "Allotriophagy"[all] OR "Geophagia"[all] OR "Rumination Syndrome*"[all] OR "Merycism"[all] OR "Rumination Disorder*"[all] OR "Anorexia Nervosa*"[all] OR (Nervosas[all] AND Anorexia[all]) OR "Bulimia Nervosa"[all] OR (Nervosa[all] AND Bulimia[all]) OR "Bulimia*"[all] OR "Binge Eating"[all] OR "Binge Eating Disorder*"[all] OR (Eating[all] AND Binge[all]) OR "Hyperphagia"[all] OR "Overeating"[all] OR "Polyphagia*"[all] OR "Diabulimia"[all] OR "Binge-Eating Disorders"[all] OR (Disorder*[all] AND "Binge-Eating"[all])))  3. 1 AND 2 |
| --- | --- |
| Scopus (n=831) | 1. ALL ( "Glucagon-Like Peptide 1" ) OR ALL ( "Glucagon Like Peptide 1" ) OR ALL ( "GLP-1" ) OR ALL ( "GLP 1" ) OR ALL ( "Glucagon-Like Peptide-1" ) OR ALL ( "Liraglutide" ) OR ALL ( "NN 2211" ) OR ALL ( "NN2211" ) OR ALL ( "NN-2211" ) OR ALL ( 2211 AND nn ) OR ALL ( "exenatide" ) OR ALL ( "glucagonlike peptide-1 receptor" ) OR ALL ( "glucagon-like peptide-1 receptor agonist" ) OR ALL ( "lixisenatide" ) OR ALL ( "semaglutide" ) OR ALL ( "albiglutide" ) OR ALL ( "dulaglutide" ) OR ALL ( "Glucagon-Like Peptides" ) OR ALL ( "Glucagon Like Peptides" ) OR ALL ( polypeptide AND "Glucagon-Like" ) OR ALL ( "Glucagon-Like Polypeptides" ) OR ALL ( "Glucagon Like Polypeptide*" ) OR ALL ( peptide AND "Glucagon-Like" ) OR ALL ( "Gut Glucagon" ) OR ALL ( "Enteroglucagons" ) OR ALL ( glucagon AND gut ) OR ALL ( "Glucagon-Like Peptide 2" ) OR ALL ( "Glucagon Like Peptide 2" ) OR ALL ( "Proglucagon (126-158)" ) OR ALL ( "Glucagon-Like Peptide-2 (1-33)" ) OR ALL ( "WB4-24" ) OR ALL ( "polyethylene glycol loxenatide" ) OR ALL ( "PEX168" ) OR ALL ( "GW002 peptide" ) OR ALL ( "rGLP-1" ) OR ALL ( "CNTO 736" ) OR ALL ( "CNTO-736" ) OR ALL ( "S 23521" ) OR ALL ( "(a8-des R36) GLP-1-(7-37)-NH2" ) OR ALL ( "AQVE-10010" ) OR ALL ( "ZP10A peptide" ) OR ALL ( "ZP 10" ) OR ALL ( "ZP-10" ) OR ALL ( "glucagon-like peptide 1 (7-36)amide" ) OR ALL ( "GLP-1-(7-36) amide" ) OR ALL ( "GLP-1a" ) OR ALL ( "proglucagon (78-107) amide" ) OR ALL ( "GLP-I (7-36)NH2" ) OR ALL ( "glucagon-like peptide-I (7-36)amide" ) OR ALL ( "glucagon-like peptide-1(7-36 amide)" ) OR ALL ( "GLP-1 (7-36)amide" ) OR ALL ( "GLP-I (7-36)amide" ) OR ALL ( "MKC 253" ) OR ALL ( "MKC253" ) OR ALL ( "MKC-253" ) OR ALL ( "GLP-1(32-36)amide" ) OR ALL ( "BMS 686117" ) OR ALL ( "BMS686117" ) OR ALL ( "BMS-686117" ) OR TITLE-ABS-KEY ( "Val(8)-" ) OR TITLE-ABS-KEY ( "Val8-GLP-1(7-36)" ) OR TITLE-ABS-KEY ( "LY 2189265" ) OR TITLE-ABS-KEY ( "LY-2189265" ) OR TITLE-ABS-KEY ( "LY2189265" ) OR TITLE-ABS-KEY ( "Glucagon-Like Peptide-1 Receptor" ) OR TITLE-ABS-KEY ( "Glucagon Like Peptide 1 Receptor" ) OR TITLE-ABS-KEY ( "GLP-1R Receptor" ) OR TITLE-ABS-KEY ( "GLP 1R Receptor" ) OR TITLE-ABS-KEY ( receptor AND "GLP-1R" ) OR TITLE-ABS-KEY ( "GLP1R Protein" ) OR ALL ( protein AND glp1r ) OR ALL ( "GLP-1 Receptor" ) OR ALL ( "GLP 1 Receptor" ) OR ALL ( receptor AND "GLP-1" ) OR TITLE-ABS-KEY ( receptor AND glp1r ) OR TITLE-ABS-KEY ( "Glucagon-Like Peptide Receptors" ) OR TITLE-ABS-KEY ( "Glucagon Like Peptide Receptors" ) OR TITLE-ABS-KEY ( "Peptide Receptors" ) AND TITLE-ABS-KEY ( "Glucagon-Like" ) OR TITLE-ABS-KEY ( receptors AND "Glucagon-Like Peptide" ) OR TITLE-ABS-KEY ( "GLPR Protein" ) OR TITLE-ABS-KEY ( protein AND "GLPR" ) OR TITLE-ABS-KEY ( "Glucagon-Like Receptor" ) OR TITLE-ABS-KEY ( "Glucagon Like Receptor" ) OR TITLE-ABS-KEY ( receptor AND "Glucagon-Like" ) OR TITLE-ABS-KEY ( proteins AND "GLPR" ) OR TITLE-ABS-KEY ( "Glucagon-Like Receptors" ) OR TITLE-ABS-KEY ( "Glucagon Like Receptors" ) OR TITLE-ABS-KEY ( receptors AND "Glucagon-Like" ) OR TITLE-ABS-KEY ( "Glucagon-Like Peptide Receptor" ) OR TITLE-ABS-KEY ( "Glucagon Like Peptide Receptor" ) OR TITLE-ABS-KEY ( "Peptide Receptor" ) AND TITLE-ABS-KEY ( "Glucagon-Like" ) OR TITLE-ABS-KEY ( receptor AND "Glucagon-Like Peptide" ) OR TITLE-ABS-KEY ( "Glucagon-Like Peptide-2 Receptor" ) OR TITLE-ABS-KEY ( "Peptide-2 Receptor" AND "Glucagon-Like" ) OR TITLE-ABS-KEY ( receptor AND "Glucagon-Like Peptide-2" ) OR TITLE-ABS-KEY ( "GLP-2 Receptor" ) OR TITLE-ABS-KEY ( "GLP 2 Receptor" ) OR TITLE-ABS-KEY ( receptor AND "GLP-2" ) OR TITLE-ABS-KEY ( protein AND "GLP2R" ) OR TITLE-ABS-KEY ( "Glucagon-Like Peptide 2 Receptor" ) OR TITLE-ABS-KEY ( "Glucagon Like Peptide 2 Receptor" ) OR TITLE-ABS-KEY ( receptor AND "GLP-2R" ) OR TITLE-ABS-KEY ( "Pro-Glucagon" ) OR TITLE-ABS-KEY ( "Pro Glucagon" ) OR TITLE-ABS-KEY ( "Preproglucagons" ) OR TITLE-ABS-KEY ( "Pre-Proglucagons" ) OR TITLE-ABS-KEY ( "Glucagon Precursor*" ) )  2. ALL ( "Feeding and Eating Disorders" ) OR ALL ( "Eating and Feeding Disorders" ) OR TITLE-ABS-KEY ( disorder* AND feeding ) OR TITLE-ABS-KEY ( "Feeding Disorder*" ) OR TITLE-ABS-KEY ( disorder* AND eating ) OR TITLE-ABS-KEY ( "Eating Disorder*" ) OR TITLE-ABS-KEY ( "Appetite Disorders" ) OR TITLE-ABS-KEY ( "Feeding and Eating Disorders of Childhood" ) OR TITLE-ABS-KEY ( "Childhood Eating and Feeding Disorders" ) OR TITLE-ABS-KEY ( "Food Addiction*" ) OR TITLE-ABS-KEY ( "Compulsive Eating" ) OR TITLE-ABS-KEY ( eating AND compulsive ) OR TITLE-ABS-KEY ( "Night Eating Syndrome*" ) OR TITLE-ABS-KEY ( "Eating Syndromes" AND night ) OR TITLE-ABS-KEY ( "Orthorexia Nervosa" ) OR TITLE-ABS-KEY ( nervosa AND orthorexia ) OR TITLE-ABS-KEY ( "Orthorexia" ) OR TITLE-ABS-KEY ( "Obsessive Healthy Eating" ) OR TITLE-ABS-KEY ( healthy AND eating AND obsessive ) OR TITLE-ABS-KEY ( "Pica" ) OR TITLE-ABS-KEY ( "Allotriophagy" ) OR TITLE-ABS-KEY ( "Geophagia" ) OR TITLE-ABS-KEY ( "Rumination Syndrome" ) OR TITLE-ABS-KEY ( "Rumination Syndrome*" ) OR TITLE-ABS-KEY ( "Merycism" ) OR TITLE-ABS-KEY ( "Rumination Disorder*" ) OR TITLE-ABS-KEY ( "Rumination Disorder" ) OR TITLE-ABS-KEY ( "Nervosa" ) OR TITLE-ABS-KEY ( "Anorexia Nervosas" ) OR TITLE-ABS-KEY ( nervosas AND anorexia ) OR TITLE-ABS-KEY ( "Bulimia Nervosa" ) OR TITLE-ABS-KEY ( nervosa AND bulimia ) OR TITLE-ABS-KEY ( "Bulimia*" ) OR TITLE-ABS-KEY ( "Binge Eating" ) OR TITLE-ABS-KEY ( "Binge Eating Disorders" ) OR TITLE-ABS-KEY ( eating AND binge ) OR TITLE-ABS-KEY ( "Hyperphagia" ) OR TITLE-ABS-KEY ( "Overeating" ) OR TITLE-ABS-KEY ( "Polyphagia*" ) OR TITLE-ABS-KEY ( "Diabulimia" ) OR TITLE-ABS-KEY ( "Binge Eating Disorder" ) OR TITLE-ABS-KEY ( "Binge-Eating Disorders" ) OR TITLE-ABS-KEY ( disorders AND binge-eating ))  3. 1 AND 2 |
| Web of Sciences  (n=1605) | 1."Feeding and Eating Disorders" (All) OR "Eating and Feeding Disorders" (All) OR (disorder* AND feeding ) (Topic) OR "Feeding Disorder*" (Topic) OR (disorder* AND eating ) (Topic) OR "Eating Disorder*" (Topic) OR "Appetite Disorders" (Topic) OR "Feeding and Eating Disorders of Childhood" (Topic) OR "Childhood Eating and Feeding Disorders" (Topic) OR "Food Addiction*" (Topic) OR "Compulsive Eating" (Topic) OR (eating AND compulsive ) (Topic) OR "Night Eating Syndrome*" (Topic) OR ("Eating Syndromes" AND night) (Topic) OR "Orthorexia Nervosa" (Topic) OR (nervosa AND orthorexia) (Topic) OR "Orthorexia" (Topic) OR "Obsessive Healthy Eating" (Topic) OR (healthy AND eating AND obsessive) (Topic) OR "Pica" (Topic) OR "allotrophagia" (Topic) OR "Geophagia" (Topic) OR "Rumination Syndrome" (Topic) OR "Rumination Syndrome*" (Topic) OR "merycism" (Topic) OR "Rumination Disorder*" (Topic) OR "Nervosa" (Topic) OR "Anorexia nervosus" (Topic) OR (nervosus AND anorexia ) (Topic) OR "Bulimia Nervosa" (Topic) OR (nervosa AND bulimia) (Topic) OR "Bulimia*" (Topic) OR "Binge Eating" (Topic) OR (eating AND binge ) (Topic) OR "Hyperphagia" (Topic) OR "Overeating" (Topic) OR "Polyphagia*" (Topic) OR "Diabulimia" (Topic) OR "Binge Eating Disorder*" (Topic) OR "Binge-Eating Disorder*" (Topic) OR (disorders AND binge-eating) (Topic)  **2. "Glucagon-Like Peptide 1"** (Topic) **OR "Glucagon Like Peptide 1"** (Topic) **OR "GLP-1"** (Topic) **OR "GLP 1"** (Topic) **OR "Glucagon-Like Peptide-1"** (Topic) **OR "Liraglutide"** (Topic) **OR "NN 2211"** (Topic) **OR "NN2211"** (Topic) **OR "NN-2211"** (Topic) **OR "2211 AND NN"** (Topic) **OR "exenatide"** (Topic) **OR "glucagonlike peptide-1 receptor"** (Topic) **OR "glucagon-like peptide-1 receptor agonist"** (Topic**) OR "lixisenatide"** (Topic) **OR "semaglutide"** (Topic) **OR "albiglutide"** (Topic) **OR "dulaglutide"** (Topic) **OR "Glucagon-Like Peptides"** (Topic**) OR "Glucagon Like Peptides"** (Topic) **OR (polypeptide AND "Glucagon-Like")** (Topic) **OR "Glucagon-Like Polypeptides"** (Topic) **OR "Glucagon Like Polypeptide*"** (Topic) **OR (peptide AND "Glucagon-Like" )** (Topic) **OR "Gut Glucagon"** (Topic) **OR "enteroglucagon"** (Topic) **OR (glucagon AND gut )** (Topic) **OR "Glucagon-Like Peptide 2"** (Topic) **OR "Glucagon Like Peptide 2"** (Topic) **OR "Proglucagon (126-158)"** (Topic) **OR "Glucagon-Like Peptide-2 (1-33)"** (Topic) **OR "WB4-24"** (Topic) **OR "polyethylene glycol loxenatide"** (Topic) **OR "pex14p"** (Topic) **OR "GW002 peptide"** (Topic) **OR "rGLP-1"** (Topic) **OR "CNTO 736"** (Topic) **OR "CNTO-736"** (Topic) **OR "S 23521"** (Topic) **OR "(a8-des R36) GLP-1-(7-37)-NH2"** (Topic) **OR "AQVE-10010"** (Topic) **OR "ZP10A peptide"** (Topic) **OR "ZP 10"** (Topic) **OR "ZP-10"** (Topic) **OR "glucagon-like peptide 1 (7-36)amide"** (Topic) **OR "GLP-1-(7-36) amide"** (Topic) **OR "GLP-1a"** (Topic) **OR "proglucagon (78-107) amide"** (Topic) **OR "GLP-I (7-36)NH2"** (Topic) **OR "glucagon-like peptide-I (7-36)amide"** (Topic)  **OR "glucagon-like peptide-1(7-36 amide)"** (Topic) **OR "GLP-1 (7-36)amide"** (Topic) **OR "GLP-I (7-36)amide"** (Topic) **OR "MKC 253"** (Topic) **OR "mkc253"** (Topic) **OR "MKC-253"** (Topic) **OR "GLP-1(32-36)amide"** (Topic) **OR "BMS 686117"** (Topic) **OR "bms986120"** (Topic) **OR "BMS-686117"** (Topic) **OR "Val(8)-"** (Topic) **OR "Val8-GLP-1(7-36)"** (Topic) **OR "LY 2189265"** (Topic) **OR "LY-2189265"** (Topic**) OR "LY2189265"** (Topic) **OR "Glucagon-Like Peptide-1 Receptor"** (Topic) **OR "Glucagon Like Peptide 1 Receptor"** (Topic) **OR "GLP-1R Receptor"** (Topic) **OR "GLP 1R Receptor"** (Topic) **OR (receptor AND "GLP-1R" )** (Topic) **OR "GLP1R Protein"** (Topic) **OR (protein AND glp1r )** (Topic) **OR "GLP 1 Receptor"** (Topic) **OR (receptor AND "GLP-1")** (Topic) **OR (receptor AND glp1r )** (Topic) **OR "Glucagon-Like Peptide Receptors"** (Topic) **OR "Glucagon Like Peptide Receptors"** (Topic) **OR "Peptide Receptors"** (Topic) **OR "Glucagon-Like"** (Topic) **OR (receptors AND "Glucagon-Like Peptide")** (Topic) **OR "glpr Protein"** (Topic) **OR (protein AND "gdpr" )** (Topic) **OR "Glucagon-Like Receptor"** (Topic) **OR "Glucagon Like Receptor"** (Topic) **OR (receptor AND "Glucagon-Like" )** (Topic) **OR (proteins AND "glpr" )** (Topic) **OR "Glucagon-Like Receptors"** (Topic) **OR "Glucagon Like Receptors"** (Topic) **OR (receptors AND "Glucagon-Like" )** (Topic) **OR "Glucagon-Like Peptide Receptor"** (Topic) **OR "Glucagon Like Peptide Receptor"** (Topic) **OR "Peptide Receptor"** (Topic) **OR "Glucagon-Like"** (Topic) **OR (receptor AND "Glucagon-Like Peptide"** (Topic) **OR "Glucagon-Like Peptide-2 Receptor"** (Topic) **OR ("Peptide-2 Receptor" AND "Glucagon-Like")** (Topic) **OR (receptor AND "Glucagon-Like Peptide-2" )** (Topic) **OR "GLP-2 Receptor"** (Topic) **OR "GLP 2 Receptor"** (Topic) **OR (receptor AND "GLP-2" )** (Topic) **OR (protein AND "glp1r" )** (Topic) **OR "Glucagon-Like Peptide 2 Receptor" )** (Topic) **OR "Glucagon Like Peptide 2 Receptor"** (Topic) **OR (receptor AND "GLP-2R" )** (Topic) **OR "Pro-Glucagon"** (Topic) **OR "Pro Glucagon"** (Topic) **OR "preproglucagon"** (Topic) **OR "Pre-Proglucagons"** (Topic) **OR "Glucagon Precursor*"** (Topic)  3.1 AND 2 |
| Cochrane  (n=91) | Search Name: finally  Last Saved: 10/09/2023 01:43:27  Comment:  IDSearch  #1"GLP-1"  #2"Glucagon Like Peptide 1"  #3"NN 2211"  #4"NN2211"  #5"NN-2211"  #6"2211, NN"  #7"glucagonlike peptide-1 receptor"  #8"glucagon-like peptide-1 receptor agonist"  #9lixisenatide  #10semaglutide  #11albiglutide  #12dulaglutide  #13"Glucagon-Like Polypeptides"  #14"Gut Glucagon"  #15Enteroglucagons  #16"WB4-24"  #17"polyethylene glycol loxenatide"  #18PEX168  #19"GW002 peptide"  #20"(a8-des R36) GLP-1-(7-37)-NH2"  #21"S 23521"  #22"CNTO-736"  #23"rGLP-1"  #24"AQVE-10010"  #25"ZP10A peptide"  #26"ZP 10"  #27"glucagon-like peptide 1 (7-36)amide"  #28"GLP-1-(7-36) amide"  #29"GLP-1a"  #30"proglucagon (78-107) amide"  #31"GLP-I (7-36)NH2"  #32"glucagon-like peptide-I (7-36)amide"  #33"glucagon-like peptide-1(7-36 amide)"  #34"MKC 253"  #35"GLP-1(32-36)amide"  #36"BMS 686117"  #37"Val(8)-"  #38"Val8-GLP-1(7-36)"  #39"LY 2189265"  #40"GLP 1R Receptor"  #41Receptor, GLP-1R  #42"GLP1R Protein"  #43"GLP-1R Receptor"  #44"GLP-1 Receptor"  #45Receptor, GLP-1  #46"Glucagon-Like Peptide Receptors"  #47"Glucagon Like Peptide Receptors"  #48"GLPR Protein"  #49"Glucagon-Like Receptor"  #50Proteins, GLPR  #51"Glucagon Like Peptide Receptor"  #52"GLP-2 Receptor"  #53Peptide-2 Receptor, Glucagon-Like  #54"Glucagon-Like Peptide 2 Receptor"  #55Protein, GLP2R  #56"Pre-Proglucagons"  #57"Preproglucagons"  #58"Glucagon-Like Peptide-2"  #59Receptor, GLP-2  #60MeSH descriptor: [Glucagon-Like Peptide 1] explode all trees  #61MeSH descriptor: [Liraglutide] explode all trees  #62MeSH descriptor: [Exenatide] explode all trees  #63MeSH descriptor: [Glucagon-Like Peptides] explode all trees  #64MeSH descriptor: [Glucagon-Like Peptide 2] explode all trees  #65MeSH descriptor: [Proglucagon] explode all trees  #66MeSH descriptor: [Glucagon-Like Peptide-1 Receptor] explode all trees  #67MeSH descriptor: [Glucagon-Like Peptide-2 Receptor] explode all trees  #68#1 OR #2 OR #3 OR #4 OR #5 OR #6 OR #7 OR #8 OR #9 OR #10 OR #11 OR #12 OR #13 OR #14 OR #15 OR #16 OR #17 OR #18 OR #19 OR #20 OR #21 OR #22 OR #23 OR #24 OR #25 OR #26 OR #27 OR #28 OR #29 OR #29 OR #30 OR #31 OR #32 OR #33 OR #34 OR #35 OR #36 OR #37 OR #38 OR #39 OR #40 OR #41 OR #42 OR #43 OR #44 OR #45 OR #46 OR #47 OR #48 OR #49 OR #50 OR #51 OR #52 OR #53 OR #54 OR #55 OR #56 OR #57 OR #58 OR #59 OR #60 OR #61 OR #62 OR #63 OR #64 OR #65 OR #66 OR #67  #69"Eating and Feeding Disorders"  #70Disorder, Feeding  #71"Feeding Disorder"  #72MeSH descriptor: [Feeding and Eating Disorders] explode all trees  #73Disorder, Eating  #74"Eating Disorder"  #75"Appetite Disorders"  #76"Feeding and Eating Disorders of Childhood"  #77"Childhood Eating and Feeding Disorders"  #78"Food Addiction"  #79"Compulsive Eating"  #80Eating, Compulsive  #81"Night Eating Syndrome"  #82"Eating Syndromes, Night"  #83MeSH descriptor: [Food Addiction] explode all trees  #84"Orthorexia Nervosa"  #85"Orthorexia"  #86"Obsessive Healthy Eating"  #87"Healthy Eating, Obsessive"  #88MeSH descriptor: [Orthorexia Nervosa] this term only  #89"Pica"  #90"Allotriophagy"  #91"Geophagia"  #92"Rumination Syndrome"  #93"Merycism"  #94"Rumination Disorder"  #95"Binge Eating"  #96Overeating  #99Polyphagia  #100MeSH descriptor: [Anorexia Nervosa] this term only  #101MeSH descriptor: [Anorexia Nervosa] this term only  #102MeSH descriptor: [Feeding and Eating Disorders of Childhood] this term only  #103MeSH descriptor: [Night Eating Syndrome] this term only  #104MeSH descriptor: [Night Eating Syndrome] this term only  #105MeSH descriptor: [Pica] this term only  #106MeSH descriptor: [Relative Energy Deficiency in Sport] this term only  #107MeSH descriptor: [Rumination Syndrome] this term only  #108MeSH descriptor: [Anorexia Nervosa] this term only  #109MeSH descriptor: [Bulimia Nervosa] this term only  #110MeSH descriptor: [Bulimia] this term only  #111MeSH descriptor: [Binge-Eating Disorder] this term only  #112MeSH descriptor: [Hyperphagia] explode all trees  #113MeSH descriptor: [Diabulimia] this term only  #114MeSH descriptor: [Binge-Eating Disorder] this term only  #115#76 OR #77 OR #78 OR #79 OR #80 OR #81 OR #82 OR #83 OR #84 OR #85 OR #86 OR #87 OR #88 OR #89 OR #90 OR #91 OR #92 OR #93 OR #94 OR #95 OR #96 OR #97 OR #98 OR #99 OR #100 OR #101 OR #102 OR #103 OR #104 OR #104 OR #105 OR #106 OR #107 OR #108 OR #109 OR #110 OR #111 OR #112 OR #113 OR #114  #116#68 AND #155 |
