# Supplementary material for: The impact of glucagon-like peptide-1 (GLP-1) agonists in the treatment of eating disorders: a systematic review and meta-analysis
Source: Eat Weight Disord. 2025 Feb 1;30(1):10. doi: 10.1007/s40519-025-01720-9 (PMC11787217; doi:10.1007/s40519-025-01720-9)
Supplement: Supplementary file 2 — Supplementary materials 2. [file 40519_2025_1720_MOESM2_ESM.doc]

***Supplementary Table 2.*** *STROBE checklist for observational studies and* Cochran’s risk of bias for clinical trials

| **Items of STROBE checklist** | **Title and abstract** | | **Background/rationale** | **Objectives** | **Study design** | **Setting** | **Participants** | | **Variables** | **Data sources/ measurement** | **Bias** | **Study size** | **Quantitative variables** | **Statistical methods** | | | | | **Participants** | | | **Descriptive data** | | | **Outcome data** | **Main results** | | | **Other analyses** | **Key results** | **Limitations** | **Interpretation** | **Generalizability** | **Funding** | **Average** |
| --- | --- | --- | --- | --- | --- | --- | --- | --- | --- | --- | --- | --- | --- | --- | --- | --- | --- | --- | --- | --- | --- | --- | --- | --- | --- | --- | --- | --- | --- | --- | --- | --- | --- | --- | --- |
| **Papers by author** | a | b |  |  |  |  | a | b |  |  |  |  |  | a | b | c | d | e | a | b | c | a | b | c |  | a | b | c |  |  |  |  |  |  |  |
| **J. Richards et al.** | 1 | 1 | 1 | 1 | 1 | 1 | 1 | - | 1 | 1 | 0 | 0 | 1 | 1 | 0 | - | - | 0 | 1 | - | - | 1 | 0 | - | 1 | 1 | 0 | 1 | 0 | 1 | 0 | 1 | 1 | 1 | 0.71 |

| **Overall** | **Other Bias** | **Incomplete Outcome Data** | **Blinding of Outcome Assessment** | **Blinding of Participants and Personnel** | **Selective Reporting** | **Allocation Concealment** | **Random Sequence Generation** |  |
| --- | --- | --- | --- | --- | --- | --- | --- | --- |
|  |  |  |  |  |  |  |  | **K. C. Allison et al.** |
|  |  |  |  |  |  |  |  | **A. Da Porto et al.** |
|  |  |  |  |  |  |  |  | **S. A. Robert** |

**Good quality: All criteria met (i.e. low for each domain)**

**Fair quality: One criterion not met (i.e. high risk of bias for one domain) or two criteria unclear**

**Poor quality: Two or more criteria listed as high or unclear risk of bias**
