# Supplementary figures and images for: The impact of glucagon-like peptide-1 (GLP-1) agonists in the treatment of eating disorders: a systematic review and meta-analysis
Source: Eat Weight Disord. 2025 Feb 1;30(1):10. doi: 10.1007/s40519-025-01720-9 (PMC11787217; doi:10.1007/s40519-025-01720-9)

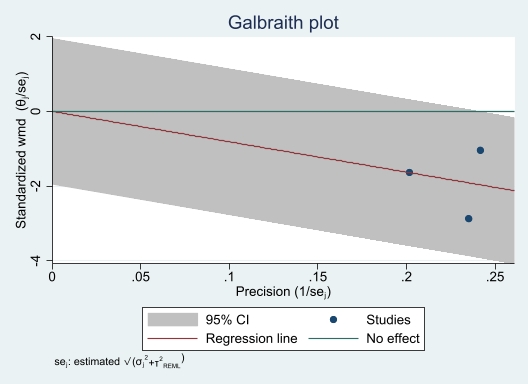

Supplement: Supplementary file 3 — Supplementary materials 3. Figure S1: Gallbraith plot assessing the potential outliers among studies comparing BES score in patients receiving GLP-1 agonists compared to control group. [file 40519_2025_1720_MOESM3_ESM.jpg]
